# Novel technique for the repair of recurrent oronasal fistula using autologous auricular composite graft: Clinical outcomes from a case series

**DOI:** 10.1016/j.jpra.2025.10.030

**Published:** 2025-10-25

**Authors:** Masanobu Yamashita, Miyuki Kishibe, Mikio Yagishita, Takayoshi Kaneko, Toru Miyanaga, Kenichi Shimada

**Affiliations:** Department of Plastic and Reconstructive Surgery, Kanazawa Medical University, 1-1 Daigaku, Uchinada, Kahoku, Ishikawa 920-0293, Japan

**Keywords:** Oronasal fistula, Cleft palate, Composite graft, Auricular cartilage, Reconstructive surgery

## Abstract

**Background:**

Oronasal fistulas are common complications of cleft palate surgery. The treatment of recurrent oronasal fistulas is challenging. Several techniques have been reported for the closure of oronasal fistulas. The fistula closure rate varies according to the method used. This study introduces a novel technique using autologous auricular skin-cartilage composite grafts for the treatment of recurrent oronasal fistulas and evaluates the clinical outcomes.

**Methods:**

Thirteen patients with recurrent oronasal fistulas who had previously undergone unsuccessful fistula repair were treated using composite grafts harvested from the auricular concha. Surgical outcomes, complications, and donor site morbidity rates were retrospectively reviewed.

**Results:**

Complete closure was achieved in nine of the 13 cases (69 %) using a single procedure. Three patients underwent repeat surgery, resulting in closure in two additional cases. The overall fistula closure rate was 85 %. No significant donor-site morbidity or postoperative palatal deformities were observed. Minor residual fistulas in two patients were successfully closed during subsequent alveolar bone grafting.

**Conclusion:**

Auricular skin-cartilage composite grafting is a promising option for the management of recurrent oronasal fistulas. This technique provides reliable structural support and mucosal integration with minimal donor site complications. It may be considered as a first-line approach in selected refractory cases, although further studies are needed to confirm its long-term outcomes.

## Introduction

Oronasal fistulas are a well-known complication of cleft palate surgery. Its incidence varies widely, ranging from approximately 2.4 % to over 30 %,^1^, ^2^, ^3^, ^4^ depending on the report. It is considered to be influenced by multiple factors, including the type of cleft, the surgical procedure performed during the initial surgery, and the surgeon's level of expertise. Oronasal fistulas can have a significant impact on daily life, causing food leakage into the nasal cavity, voice hypernasality, speech disorders, and recurrent nasopharyngeal infections.^5^

Various fistula closure techniques have been reported,^6^ including local mucosal, buccal mucosal, tongue, and free skin flaps. However, each method has limitations, particularly in cases involving a history of multiple surgeries and severe scarring, in which the risk of recurrence or incomplete closure is high, highlighting the challenges of treatment. For recurrent and refractory palatal fistulas, a new treatment method that ensures adequate coverage and support is urgently required.

We developed a new closure technique for refractory fistulas using autologous auricular skin-cartilage composite tissue. In this method, skin and cartilage are harvested integrally from the auricle and transplanted into the fistula site, thereby achieving structural support and epithelial coverage. This approach is expected to provide stable closure, even in scarred or poorly vascularized areas.

## Patients and methods

This observational retrospective study included patients who underwent palatoplasty for cleft palate at the Department of Plastic and Reconstructive Surgery, Kanazawa Medical University Hospital, between January 2021 and December 2024, and who had previously undergone fistula closure surgery that resulted in recurrence. Patients with velopharyngeal insufficiency were excluded.

This study was approved by the Institutional Review Board of Kanazawa Medical University (C207) and complied with the Declaration of Helsinki (2024).

### Surgical technique

The procedure was performed under general anesthesia under loupe magnification. Local anesthesia was administered using a 0.5 % lidocaine solution containing epinephrine. Under optimal conditions, the mucosa around the fistula was elevated as a mucoperiosteal flap and inverted as a turnover flap to create a lining on the nasal cavity side. The mucoperiosteal layer around the fistula was dissected toward the lateral side of the fistula to create a submucosal pocket. The pocket size was determined based on the size of the cartilage to be harvested.

**Figure 1:**
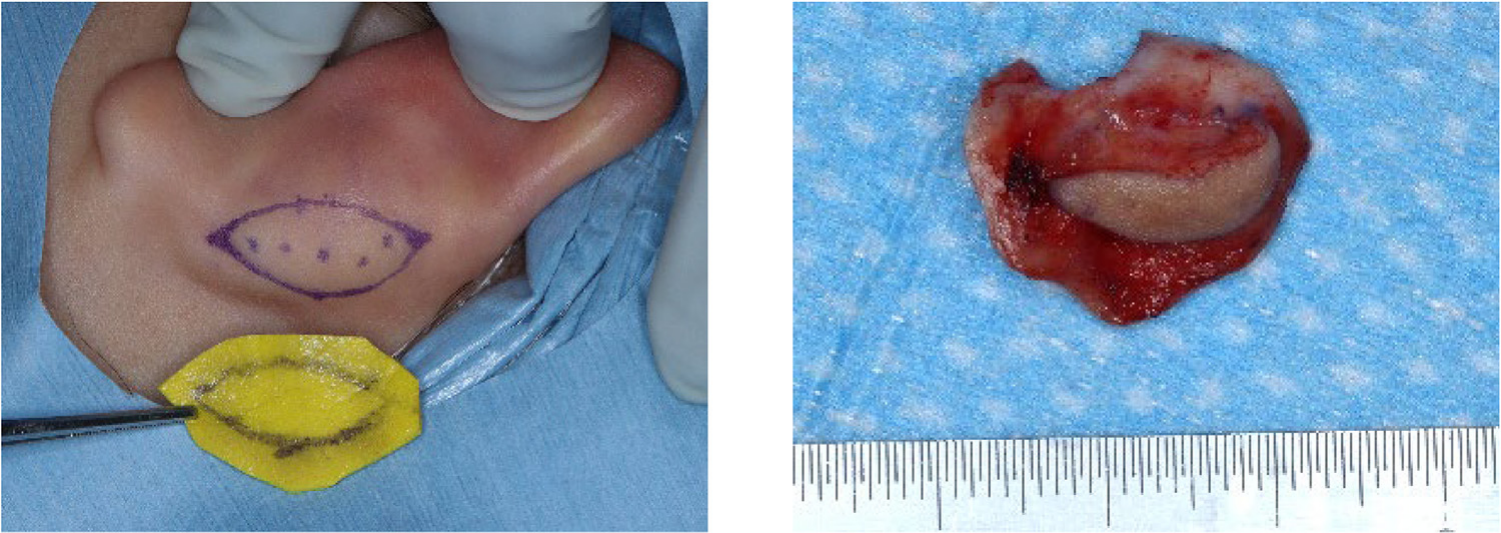
A composite graft including the skin of the posterior surface of the auricle is harvested.

**Figure 2:**
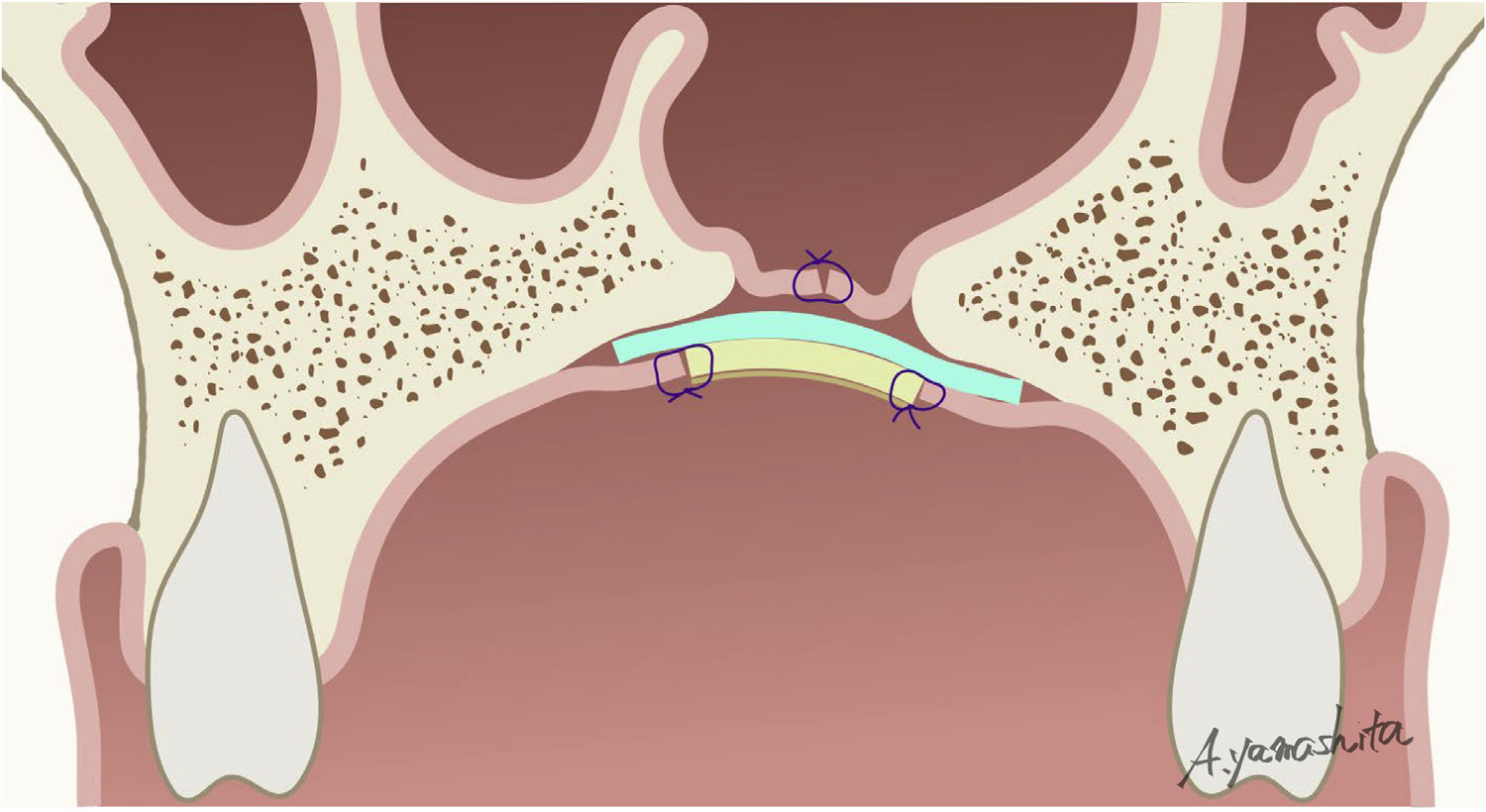
Coronal section of an oronasal fistula. Two mucoperiosteal flaps are elevated to create a lining on the nasal cavity side. The auricular composite graft is transplanted, and the skin is sutured firmly to the surrounding palatal mucosa without tension.

**Figure 3:**
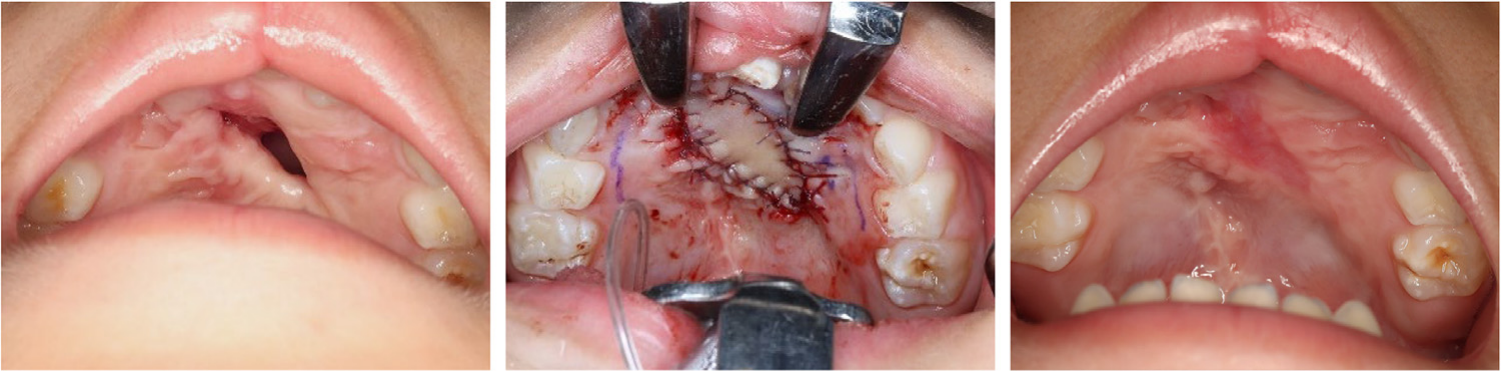
Preoperative (left); an auricular composite graft is employed for the repair of Pittsburgh type V fistula (center); and 2 months postoperatively (right).

The composite graft was then harvested from the auricular concha. In this procedure, the auricular cartilage is harvested with the perichondrium attached to both the anterior and posterior surfaces. The skin paddle was removed from the posterior skin of the auricle (Figure 1), and its size was determined by the extent of the mucosal defect on the oral side. The harvested skin was sutured, and tie-over fixation was performed to prevent hematoma formation. The cartilaginous portion of the auricular composite tissue was inserted into the submucosal pocket around the fistula. Pull-out sutures were unnecessary. Finally, the surrounding oral mucosa and skin were sutured with 5–0 polyglactin 910 sutures to achieve watertight closure (Figure 2, Figure 3). No special dressing was required at the wound site. Soft food intake was initiated on the day after surgery.

## Results

This procedure was performed in 13 patients with recurrent oronasal fistulas. Ten male and three female patients were included. The average age of the patients was 12 years (range, 5–26 years). The follow-up period ranged from 7 to 54 months, with an average of 29 months. The patient data are shown in Table 1. The primary cleft was Veau II in three cases, Veau III in seven cases, and Veau IV in three cases. The location of the fistula was classified as Type IV (hard palate) in nine cases and Type V (border between the primary and secondary palate) in four cases, according to the Pittsburgh classification.^7^ The length of the fistulas ranged from 6 to 14 mm (mean, 10 mm), and their widths varied from 5 to 10 mm (mean, 7 mm). The average number of fistula closure procedures performed prior to the current procedure was two (range: 1–4).

**Table 1:** Demographic and clinical characteristics of the study population.

Case	Age (years)	Sex	Size of fistula^b^ (mm)	Veau classification	Pittsburg classification	Number of previous fistula procedures	Fistula recurrence after 1st AACG	Fistula Recurrence after 2nd AACG
1	14	M	8 × 8	III	V	4	No	
2	5	M	10 × 6	IV	V	1	No	
3	5	M	12 × 8	III	V	1	Yes	Yes^a^
4	10	M	10 × 10	II	IV	2	No	
5	18	F	10 × 5	III	IV	2	No	
6	13	M	10 × 5	III	IV	1	No	
7	5	M	14 × 8	IV	IV	4	Yes	No
8	26	M	10 × 7	III	IV	4	No	
9	13	F	10 × 9	II	IV	2	No	
10	15	M	8 × 6	IV	IV	2	No	
11	8	M	8 × 6	III	IV	1	Yes	No
12	17	F	6 × 6	II	IV	2	No	
13	12	F	14 × 8	III	IV	3	Yes^a^	–

AACG, Autologous auricular composite graft.

a Subsequent closed with alveolar bone graft.

b Largest diameter × width.

**Figure 4:**
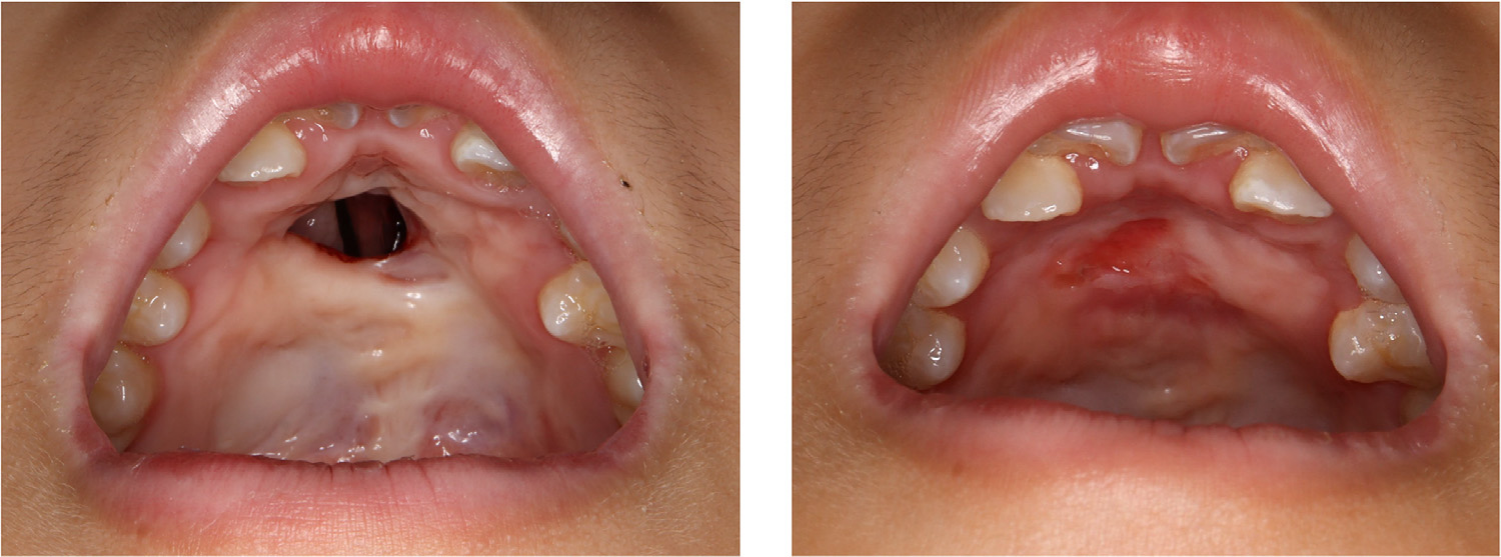
Pittsburg IV fistula. Preoperative (left) and 3 month-postoperative (right) photos of the autologous auricular composite graft.

**Figure 5:**
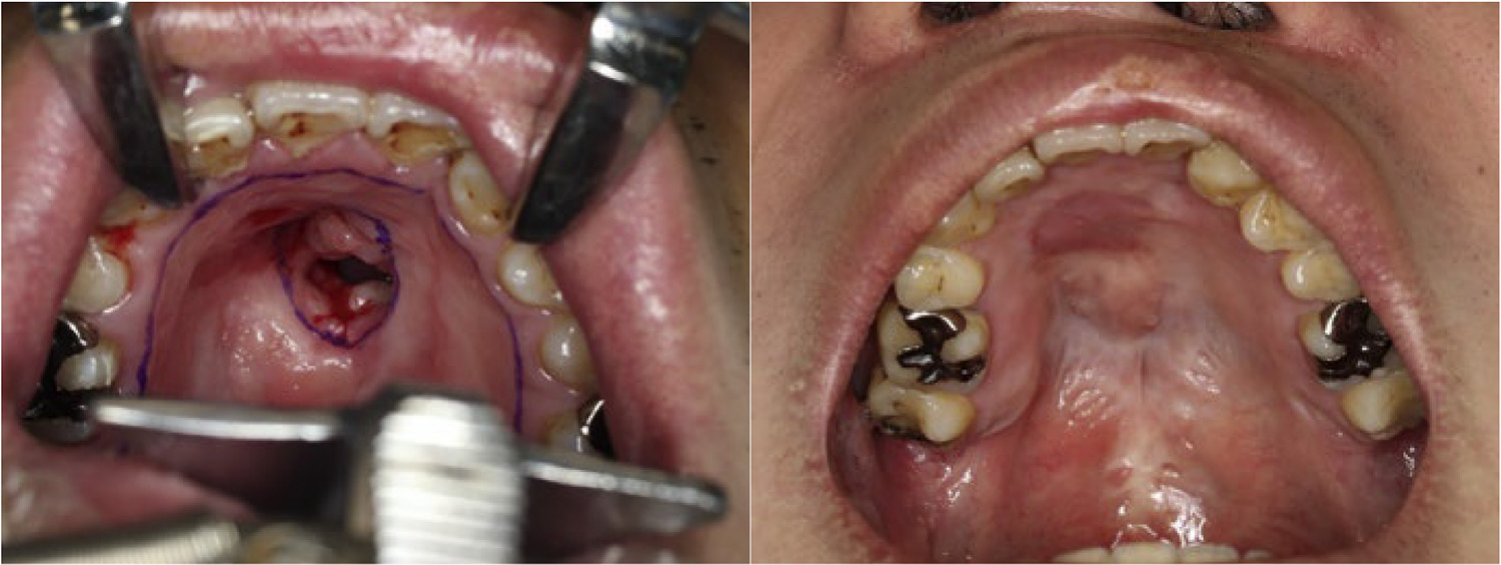
Pittsburg IV fistula. Preoperative (left) and 4 month-postoperative (right) photos of the autologous auricular composite graft.

In nine cases (69 %), the fistula was completely closed. In four cases (31 %), the fistula recurred postoperatively. Among the four recurrence cases, the same procedure was repeated in three, resulting in closure of the fistula in two cases and a residual small fistula on the lingual side of the alveolar in one case. The complete fistula closure rate using this method, including that in cases with multiple surgeries, was 85 % (11/13 cases). After fistula closure, the palatal morphology maintained a normative structure with minimal irregularities or deformities in the surface mucosa, and no deformities or irregularities commonly observed with other methods were indicated (Figure 4, Figure 5). In two cases where small fistulas persisted, they were subsequently closed using alveolar bone grafting. No obvious auricular deformities were observed after composite tissue harvesting. No postoperative nasopharyngeal closure dysfunction has been reported.

## Discussion

Palatal fistulas are common complications following cleft palate surgery, causing various functional disorders, such as speech impairment, nasal discharge, and difficulty in eating. Reconstruction is complex because of scarring, thinning of the tissue surrounding the fistula, poor blood flow, and deterioration of the local environment caused by repeated surgeries. Various closure techniques have been proposed, each with its advantages and disadvantages, and no established standard procedure exists.

The most commonly used traditional technique is the use of a local mucosal flap (palatal mucosal flap).^8^, ^9^, ^10^, ^11^ This method is relatively minimally invasive and easy to perform. It is considered appropriate for primary closure surgery of small fistulas with good mucosal conditions around the fistula. However, this often results in excessive tension sutures due to scarring and insufficient tissue volume around the fistula, leading to a high risk of fistula recurrence. Closures using only a single-layer turnover flap have a high recurrence rate of palatal fistulas, with a recurrence rate of approximately 40 %.^12^ For reliable fistula closure, reconstruction using two layers, one on the nasal lining and the other on the oral layer, is preferable. Clinically, the turnover flap is often used for the nasal mucosal reconstruction of other flaps. A revision palatoplasty is indicated when the fistula is located in the central palate. At our institution, we primarily prefer two-flap palatoplasty.^3^ Revision palatoplasty is the first choice, especially in cases where velopharyngeal insufficiency is detected on preoperative examination. The buccal mucosal flap^13^^,^^14^ provides greater tissue volume and is considered adequate for fistulas of the posterior palate. Owing to the extensibility of the mucosa and the high mobility of the flap, viable closure can be achieved. The FAMM flap^15^^,^^16^ has favorable postoperative survival because of the stable blood flow from the facial artery. The flap was sufficiently long and flexible to reach the middle and posterior palate. Similar to a buccal mucosal flap, it can be completed using an intraoral approach, leaving no external scars. The tongue flap^17^, ^18^, ^19^ is a musculomucosal flap with rich blood flow and thickness, and provides an effective option for recurrent cases and large fistulas. It is beneficial for anterior fistulas but requires the tongue and palate to remain connected for several weeks after surgery, which significantly affects daily activities such as eating, speech, and hygiene. Therefore, their use in children should be carefully considered. During the initial postoperative period, precautions must be taken to prevent the flap from detaching due to tongue movements. A tongue flap is a two-stage procedure that is required to separate the flap. Recently, fistula closure using buccal fat pads has gained attention.^20^^,^^21^ The buccal fat pad has abundant blood flow, excellent mobility, and reconstructive capacity, making it a soft tissue that can easily accommodate the fistula site. These relatively minimally invasive harvesting techniques are advantageous. Recent reports have shown high closure success rates for small-to-moderate fistulas, particularly in cases with thin local tissue. It is recommended for fistulas located in the posterior two-thirds of the palate and with a length of 20 mm or less. Separation surgery is occasionally required.^22^ Thus, the efficacy of conventional surgical procedures varies depending on individual cases, and it is essential to determine the most suitable procedure for each patient.

Auricular composite grafting reported in our study is a new option to address these problems. This procedure involves transplanting composite tissue, including skin and cartilage, from the autologous auricle to the fistula site to integrally reconstruct both the skin and supporting tissue. The main features of this procedure are as follows: (1) it is a single-stage surgery; (2) it provides tissue with sufficient thickness and strength directly at the fistula site; (3) it may reduce the risk of postoperative contraction or depression by utilizing the elasticity and shape-retaining ability of the auricular cartilage; and (4) scarring and functional impairment at the donor site are minimized.

As previously mentioned, the recurrence rate after palatal fistula closure surgery varies depending on the study. A study by Abdali et al.^23^ reported a recurrence rate of 7 % after initial fistula closure surgery and a recurrence rate of 33 % after subsequent fistula closure surgery for recurrent fistulas. This suggests that recurrent oronasal fistulas are more challenging to treat than those occurring after initial palatoplasty.

In this case series, despite all cases exhibiting previously failed closure with conventional methods, such as local mucosal flaps or tongue flaps, relatively beneficial results were achieved. With this surgical procedure, complete closure of the fistula was achieved in 69 % of patients with a single surgery. The fistula closure rate, including that of two cases requiring two surgeries, was 85 %. None of these cases showed recurrence of fistula formation at least 7 months postoperatively. The two patients with residual fistulas were asymptomatic and had tiny fistulas that were successfully closed during subsequent alveolar bone grafting.

Previous studies described the use of autologous cartilage as a simple interpositional graft.^23^^,^^24^ In contrast, the current technique involves transplanting the cartilage as a composite tissue, including the skin, which enables secure suture fixation with minimal tension between the skin and surrounding mucosa. This is a notable difference from the previous cartilage grafting methods. Autologous skin-cartilage composite grafts are transplanted into composite tissue, creating a strong mechanical barrier that is essential for fistula closure. This robust graft may also be effective in preventing late-onset palatal fistulas that occur during subsequent orthodontic treatment with palatal expansion.

The mechanism of blood flow restoration from the posterior auricular skin stump may be involved in the healing process of autologous skin-cartilage composite grafts using this method.^25^ We encountered cases in which the transplanted skin was necrotized and detached, particularly in relatively large fistulas. In all cases, rapid wound closure was achieved within approximately 4 weeks of epithelialization. Graft take depends on the blood flow around the fistula, and areas with a history of scarring or infection may have poor graft take. Additionally, the amount of tissue that can be harvested from the auricle is limited, and caution is required when treating large fistulas. Interestingly, this method has no irregularity of the palatal mucosal surface or deformation due to flaps after palatal fistula closure, unlike those involving mucosal flaps. To preserve the typical morphology of the palate, this method has potential for both recurrent cases and as an initial surgical procedure for fistulas following cleft palate repair. Furthermore, by incorporating regenerative medicine in the future to enhance functionality, a strategized treatment for fistula closure can be established.

Composite tissue grafts containing transplantable cartilage include not only the auricular composite tissue used in this study, but also nasal septal cartilage–mucosa composite tissue and costal cartilage composite tissue. Nasal septal cartilage–mucosa composite tissue is thin and flat, making it easily adaptable to the shape of the palate. Furthermore, if the mucosa engrafts, it is likely to integrate well into the oral cavity. However, for adult patients with cleft lip and palate, it may be used during rhinoplasty. Furthermore, as patients with cleft lip and palate inherently have weak nasal septal support, preserving the nasal septal cartilage is desirable. It might be a good indication for isolated cleft palate cases. While harvesting the nasal septal cartilage composite graft is relatively easy, as only the mucosa of one side of the nasal septum remains at the donor site, care must be taken to avoid creating a septal perforation. Costal cartilage grafts are too thick to incorporate superficial skin as a skin paddle. The strong perichondrium of the costal cartilage is considered suitable for suturing to the edge of the cleft palate. Therefore, it may be possible to transplant it as a composite graft consisting of thinly sliced costal cartilage together with the attached perichondrium. Care must be taken to avoid pneumothorax during harvesting. The auricular composite tissue used in the present study was considered an excellent graft material for palatal fistula closure. However, a potential donor site issue is the possibility of auricular deformity. While we have not yet encountered cases leading to auricular deformity, from the perspective of preventing these deformities, preserving the auricular perichondrium on the auricle side during composite tissue harvesting may be preferable.

## Limitations

This was a retrospective study with a small sample size, necessitating future prospective studies with a large number of cases, which will advance our understanding and improve cleft palate treatment.

## Conclusion

Autologous auricular skin–cartilage composite grafts offer a promising new option for the treatment of recurrent oronasal fistulas following cleft palate surgery. In this case series, the technique demonstrated a high closure rate even in patients with a history of multiple failed repairs and resulted in stable, structurally sound closures with minimal donor-site morbidity. Further studies are warranted to validate these findings and explore their potential roles as first-line treatment options.

## Author contribution

M Yamashita and M Kishibe conceived and designed the study, performed surgeries, analyzed the data, and drafted the manuscript. M Yagishita, T Kaneko, and T Miyanaga contributed to patient management and data collection and interpretation and critically revised the manuscript. K Shimada supervised the study, contributed to its design, and provided critical revisions and final approval of the manuscript.

## Funding

The authors have no funding to disclose.

## Declaration of generative AI and AI-assisted technologies in the writing process

None.

## Ethical approval

This study was approved by the Institutional Review Board of Kanazawa Medical University (C207) and complied with the Declaration of Helsinki (2024).

## Informed consent

Informed consent for the publication of photographs was obtained from all patients whose images are included in this manuscript.

## Declaration of competing interest

None.
